# GRAPE‐WEB: An automated computational redesign web server for improving protein thermostability

**DOI:** 10.1002/mlf2.12152

**Published:** 2024-12-24

**Authors:** Jinyuan Sun, Wenyu Shi, Zhihui Xing, Guomei Fan, Qinglan Sun, Linhuan Wu, Juncai Ma, Yinglu Cui, Bian Wu

**Affiliations:** ^1^ AIM Center, College of Life Sciences and Technology, Beijing University of Chemical Technology, Institute of Microbiology Chinese Academy of Sciences Beijing China; ^2^ University of Chinese Academy of Sciences Beijing China; ^3^ State Key Laboratory of Animal Biotech Breeding, College of Biological Sciences China Agricultural University Beijing China; ^4^ Microbial Resource and Big Data Center, Institute of Microbiology Chinese Academy of Sciences Beijing China; ^5^ Chinese National Microbiology Data Center (NMDC) Beijing China

## Abstract

We have developed the GReedy Accumulated strategy for Protein Engineering (GRAPE) to improve enzyme stability across various applications, combining advanced computational methods with a unique clustering and greedy accumulation approach to efficiently explore epistatic effects with minimal experimental effort. To make this strategy accessible to nonexperts, we introduced GRAPE‐WEB, an automated, user‐friendly web server that allows the design, inspection, and combination of stabilizing mutations without requiring extensive bioinformatics knowledge. GRAPE‐WEB's robust performance and accessibility provide a comprehensive and adaptable approach to protein thermostability design, suitable for both newcomers and experienced practitioners in the field. The web server is accessible at https://grape.wulab.xyz.

Nature has evolved a wide array of enzymes crucial for numerous essential biochemical functions. While these natural enzymes are rarely optimal for industrial application, protein engineering can enhance their performance by improving their physical and catalytic properties^1^. However, redesigning enzymes for new functions often reduces their stability and may even cause misfolding of the target enzyme^2^. Consequently, there is a growing demand for robust enzymes that not only meet industrial requirements but also possess evolutionary potential for further optimization.

In recent decades, directed evolution has been used to successfully tailor enzyme properties, but the costly, time‐ and labor‐demanding nature of this approach makes in silico screening of protein variants a highly attractive alternative^3^. Various computational methods, including energy‐based, phylogeny‐based, and machine learning methods, have been used to design single‐point mutations for improved protein stability. However, the accuracy of individual algorithms still suffers from a series of problems, such as insufficient conformational sampling, inaccurate force fields, or imbalances in protein data sets, leading to increased prediction errors, especially for multiple‐point mutants^1^.

To address these limitations, researchers have developed hybrid methods that incorporate complementary approaches, reducing the sampling bias of individual approaches^4^. However, the resulting expanded library of beneficial mutations has made it more challenging to identify optimal combinations^5^, ^6^. When coupled mutations interact epistatically, predictions often lead to nonconvergent optimization processes, failing to achieve near‐optimal mutation combinations.

To address this challenge, we have developed the GReedy Accumulated strategy for Protein Engineering (GRAPE) to search for beneficial combination pathways in a large single‐point mutation library^7^. Leveraging hybrid methods, clustering, and greedy algorithms, GRAPE maximizes the exploration of epistatic effects while minimizing experimental efforts to identify efficient accumulation paths. The GRAPE (Figure 1) employs a hybrid design approach integrating force field‐based algorithms FoldX^8^ and Rosetta^9^, along with the statistics‐based algorithm ABACUS^10^, to design potentially stabilizing single‐point mutations. After prediction, visual inspection of the predicted mutant structures eliminates variants with known pitfalls. The refined list of candidates then undergoes experimental validation to confirm their stabilizing effects. Beneficial variants are clustered into groups based on their Δ*T*_*m*_ improvements, presumed effects, and the positions of C*α* atoms for each mutation. Post‐clustering, we recommend experimentally accumulating mutations within each cluster, following a greedy strategy to achieve desired functional outcomes.

**Figure 1:**
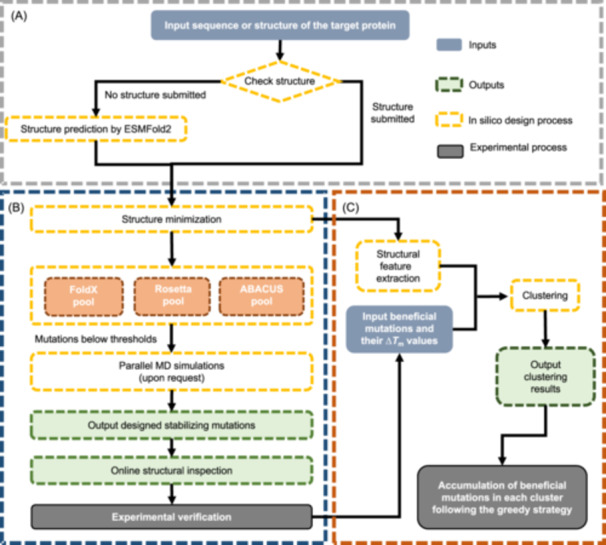
General workflow of the GReedy Accumulated strategy for Protein Engineering (GRAPE)‐WEB server. (A) Input from user. (B) Single‐mutation design. (C) Greedy accumulation.

To validate GRAPE's performance, we utilized a high‐quality data set comprising 2648 mutant variants across 131 proteins sourced from ProTherm^11^, assessed using the established PoPMuSiC method^12^, as detailed in Tables S1–S3. For benchmarking against prevalent algorithms, we selected a subset of 350 mutants from 67 distinct proteins not previously used in related algorithm training or testing. We derived ΔΔ*G*_P_ values for PoPMuSiC‐2.0, CUPSAT^13^, Dmutant^14^, Eris^15^, and Imutant^16^ from the PoPMuSiC data set^12^. Threshold values for these algorithms were based on their precision metrics. As shown in Table S4, we set thresholds for FoldX, Rosetta, and ABACUS in GRAPE‐WEB to –1.5 kcal/mol, –1.0 REU, and –2.5 AEU, respectively.

Notably, distinct mutations exclusive to each algorithm underscored their complementary roles (Table S5). Beyond mere accuracy, practical applications demand a broader set of beneficial mutations, and GRAPE, navigating through sequence space via distinct approaches, significantly expanded the repertoire of stabilizing mutations. Aiming to construct an extensive mutation library, we focused primarily on F1‐scores, encompassing both precision and recall, to ensure a broad capture of stabilizing mutations possibly overlooked by individual methods. As shown in Table 1, GRAPE demonstrated superior performance, achieving the highest F1‐score and accuracy compared to other algorithms.

**Table 1:** Comparison of GRAPE with other commonly used algorithms for predicting protein stability.

Algorithm	Threshold	Sensitivity	Specificity	Precision	Accuracy	F1‐score
PoPMuSiC‐2.0	–0.5	0.105	1.000	1.000	0.757	0.190
CUPSAT	–1.5	0.105	0.980	0.657	0.743	0.182
Dmutant	–1.5	0.200	0.957	0.633	0.751	0.304
Eris	–2.5	0.211	0.965	0.690	0.760	0.323
Imutant	–1	0.053	0.957	0.313	0.711	0.090
GRAPE	–1.5/–1/–2.5	0.389	0.906	0.607	0.766	0.474

The threshold values were chosen based on the precision results. GRAPE, GReedy Accumulated strategy for Protein Engineering.

The successful transformation of PETase from *Ideonella sakaiensis* 201‐F6 (*Is*PETase) (PDB ID: 5XH3) highlights the potential application of GRAPE for enhancing thermal stability. A library of designed single‐point mutations was obtained, with the ABACUS, Rosetta, and FoldX algorithms providing 100, 65, and 61 mutations, respectively. Additionally, consensus analysis generated 54 single‐point mutations. After structural inspection, 85 candidates were chosen for experimental validation, and 21 mutants displayed increased stability (∆*T*_*m*_ ≥ 1.5°C). We further grouped the stabilizing mutations into three clusters. The detailed clustering results are presented in Table S6. Greedy accumulation was experimentally performed to combine the single‐point mutations in each cluster. Ultimately, only 65 combined variants were explored, resulting in the most thermostable variant, referred to as DuraPETase, with significantly enhanced thermostability (Δ*T*_*m*_ = 31°C). Please refer to sections 5 and 6 in the Supporting Information section and Cui et al.^7^ for more details of this case study.

In addition to enhancing thermal stability, the GRAPE was applied to increase the organic solvent stability of a peptidylamidoglycolate lyase from *Exiguobacterium* sp.^17^. Following the design of single‐point mutations and structural analysis, 62 predicted mutants were experimentally evaluated, with 17 mutants exhibiting increased resistance to denaturing agents like guanidine hydrochloride. After clustering and greedy accumulation, a final mutant (PAL14) containing 14 mutations was obtained. PAL14 demonstrated significant improvements in denaturant tolerance, achieving a 24‐fold increase in peptide C‐terminal amidation activity under 2.5 M guanidine hydrochloride. These experimental validations underscore the efficacy of the GRAPE in augmenting enzyme robustness. Please refer to section 7 in Supporting Information section and Zhu et al.^17^ for more details of this case study.

Although GRAPE has demonstrated significant potential for enhancing protein thermostability in previous engineering projects, it is currently available only as a stand‐alone tool and requires extensive structural and bioinformatics experience to implement workflows. To address this limitation, we developed a web version of the GRAPE, GRAPE‐WEB (https://grape.wulab.xyz), for automatically improving protein thermostability. The main difference between the GRAPE‐WEB server and other stability engineering web tools is the ability to interact with the experimental results. With easy access to the online web server, nonexpert users can design potential stabilizing candidates, filter mutations with pitfalls through structural inspection, and perform clustering on the server without programming knowledge or software installation.

The web server was designed to have both “Stabilizing mutation design” and “Stabilizing mutation clustering” sections. In the “Stabilizing mutation design” section, users can initiate the process by submitting sequence or structure data for the target protein (Figure 1A). For proteins with available experimental or computationally predicted structures, submission of a PDB file is allowed. Otherwise, GRAPE‐WEB employs ESMFold^18^ to predict the structure of the target protein, sparing users the task of prediction. Users can manually choose specific chains of the target protein for design. If ESMFold is used, the chain ID must be set to “A”. The calculations allow for the application of either default or user‐defined thresholds in designing potential stabilizing mutations, with the latter offering customization for advanced users. Configuration of settings is completed by clicking the “Prepare” button, followed by job submission by clicking the “Run” button (Figure S1A). Upon submission, the single‐mutation prediction process will be launched (Figure 1B). A unique job ID will be sent to the provided email address and displayed on the screen. The “job hashrun” will be used to retrieve predicted stabilizing mutations.

Once the job is completed, users can access the “Stabilizing mutation results” section to conduct a structural analysis by replacing the demo job hash with their assigned identifier. An example provided in the demo job features the design outcomes for a limonene‐1,2‐epoxide hydrolase (PDB ID: 1NWW) (Figure S1B). Entering the user's unique identifier would reveal mutations with ABACUS energy or folding free energy (∆∆*G*_fold_) below the user‐defined thresholds. In the results table, details of the predicted mutations are presented in six columns. The initial four columns include the mutation name and its residue index, while the subsequent two columns show the utilized design algorithms and their predicted energies. Users can visualize the structure of the mutant in the Mol*^19^ viewer plugin by entering the mutation name. Within the Mol* interface, selecting the desired mutation from the sequence list using the setting button reveals its interactions with nearby amino acids, aiding in the exclusion of mutations that might lead to biophysical problems such as internal cavities, disrupted hydrogen bonds, or the exposure of hydrophobic residues, thus refining the quality of the library and minimizing screening efforts. Since all mutant structures can be directly accessed via the application programming interface (API), code snippets are provided to enable users to locally load the mutants and highlight their surroundings in PyMOL.

For users choosing the clustering step after experimental validation, the “Stabilizing mutation clustering” section (Figure 1C) requires submitting beneficial mutations and their associated ∆*T*_m_ values in either .txt or .csv format (Figure S2). Additionally, the target protein's sequence data are needed. The clustering calculations leverage features automatically generated by the server. The first three columns provide the mutation and Δ*T*_*m*_ improvements. The next three columns list the potential effects caused by the mutations, including changes in hydrogen bonding, hydrophobic interactions, and conformational entropy. The values in the “Hbond” (hydrogen bonds) and “hydrophobic” (hydrophobic interactions) columns quantify the changes in mutant interactions compared to the wild type. In the “entropy” column, only a value of 1 or 0 is displayed, where 1 indicates a potential change in entropy and 0 indicates no change. The final three columns contain the Euclidian coordinates of the C*α* atoms of the mutations.

In summary, GRAPE‐WEB is a user‐friendly web server based on the GRAPE for protein stabilization. Both in silico analysis and experimental verifications support the broad applications of this strategy. By integrating complementary methods, the GRAPE further enriches the beneficial mutation library. FoldX and Rosetta rely on energy functions to describe the strength of physical interactions between atoms. However, these methods suffer from inaccuracies in energy functions and insufficient sampling. Meanwhile, ABACUS uses a statistical energy function approach. This data‐driven method is influenced by the uneven distribution of protein structure data, potentially leading to over‐smoothing in sparsely populated regions. These methods have been shown to complement one another (Table S5). By combining these tools, GRAPE leverages their strengths, increasing the number of beneficial mutations and enriching the pool of stabilizing mutations for further combination.

There are also areas for improvement in the current methodology. While molecular dynamic (MD) simulations provide detailed insights into protein dynamics, they are too computationally intensive to be integrated into GRAPE‐WEB without significantly limiting its service capacity. Due to this limitation, GRAPE‐WEB may not perform optimally for large or highly dynamic proteins. To address this, we offer a local version of GRAPE that allows users to automate MD simulations. Additionally, advancements in mutation stability prediction using deep learning methods have shown higher success rates for predicting single mutations without MD‐based filtering^20^. However, we did not incorporate these deep learning methods into GRAPE‐WEB due to limited experimental success. It is likely that the successful design of more stable enzymes will become more accessible in the future, and we plan to optimize the combination of mutation predictors as the field progresses.

## AUTHOR CONTRIBUTIONS

**Jinyuan Sun**: Software (equal). **Wenyu Shi**: Software (equal). **Zhihui Xing**: Software (equal). **Guomei Fan**: Software (equal). **Qinglan Sun**: Software (equal). **Linhuan Wu**: Software (equal). **Juncai Ma**: Software (equal). **Yinglu Cui**: Conceptualization (equal); methodology (equal); resources (equal); software (equal). **Bian Wu**: Conceptualization (equal); supervision (equal); writing—review and editing (equal).

## ETHICS STATEMENT

Ethics statement is not applicable to this study.

## CONFLICT OF INTERESTS

The authors declare no conflict of interests.

## Supporting information

Supporting information.

## Data Availability

The data that support the findings of this study are available at https://grape.wulab.xyz/.
