# Supplementary material for: GRAPE‐WEB: An automated computational redesign web server for improving protein thermostability
Source: mLife. 2024 Dec 24;3(4):527–31. doi: 10.1002/mlf2.12152 (PMC11685837; doi:10.1002/mlf2.12152)
Supplement: Supplementary file 1 — Supporting information. [file MLF2-3-527-s001.docx]

Supplementary Materials for

**GRAPE-WEB: an automated computational redesign web server for improving protein thermostability**

Jinyuan Sun^1,2#^, Wenyu Shi^3#^, Zhihui Xing^1#^, Guomei Fan^4^, Qinglan Sun^4^, Linhuan Wu^4^, Juncai Ma^4,5^, Yinglu Cui^1*^, Bian Wu^1*^

**Affiliations:**

^1^ AIM center, College of Life Sciences and Technology, Beijing University of Chemical Technology, Institute of Microbiology, Chinese Academy of Sciences, China.

^2^ University of Chinese Academy of Sciences, Beijing, China.

^3^ State Key Laboratory of Animal Biotech Breeding, College of Biological Sciences, China Agricultural University, Beijing, China

^4^ Microbial Resource and Big Data Center, Institute of Microbiology, Chinese Academy of Sciences, Beijing 100101, China.

^5^ Chinese National Microbiology Data Center (NMDC), Beijing 100101, China

Correspondence to: [cuiyinglu@im.ac.cn](mailto:cuiyinglu@im.ac.cn) or wub@im.ac.cn

**Supplementary Methods**

**Step 1 of GRAPE strategy: Design of stabilizing mutations**

Sequence or structural data can be used as inputs for predicting stabilizing single point mutations in proteins. Prior to the design, the sequence and structural information of the protein must be verified. In the absence of an available protein structure, ESMFold is utilized to predict the structure of the submitted sequence. For crystal structures, it is important to ensure structural integrity, as breaks would cause unreasonably high energy calculations. RepairPDB of FoldX and relax of Rosetta are used to automatically complete missing atoms including hydrogens and repair residues with bad van der Waals interactions. Any ligands, cofactors, and non-standard residues are removed from the structure. The patched structure is then minimized by Rosetta_relax. Subsequently, FoldX and Rosetta and ABACUS are employed to predict potentially stabilizing point mutations. Each residue is mutated *in silico* for other 19 amino acids.

FoldX version 5 with standard settings is used, and each calculation is repeated five times to obtain an appropriate average. The GRAPE strategy applied settings described by Park et al. (1) for Rosetta_ddg. For ABACUS, ABACUS_prepare is used to calculate the residue-wise properties. ABACUS_S1S2 is subsequently used to calculate various energy terms, whereas ABACUS_singleMutationScan is used to calculate the changes of SEF energy terms related to single point mutations. After calculations by three algorithms, single mutations over a given threshold are obtained. The default values for ABACUS, FoldX, and Rosetta are -2.5 AEU, -1.5 kcal/mol and -1.0 REU, respectively. Default values are optimized to provide reliable results for most systems (Table 1 and Supplementary Tables S1-S3).

**Filtering chemically unreasonable mutations by structural inspection**

Although designed by a hybrid design approach, the predicted mutations may still exhibit unfavorable interactions due to the simplified energy function and limited conformational sampling. Structural inspection can eliminate mutations that present biophysical pitfalls, and molecular dynamics (MD) simulations can improve the filter process by identifying mutations that maintain their conformations in static structures but alter during simulations. However, given the time-consuming feature of MD simulations, GRAPE-WEB offers MD simulation services only upon specific request from users. For such requests, five parallel MD simulations, each with varying initial atom velocities, are performed for each mutant below given thresholds. The simulations are performed by OpenMM (2) with the AMBER99SB-ILDN force field and TIP-3P water model. Energy minimization step is performed to eliminate clashes, followed by an equilibration step and a 100 ps production step. For each independent MD simulation, the average structure is generated. Detailed criteria for structural inspection are shown in the Supplementary Data. Mutations that pass the structural screening should be experimentally verified by measuring ∆*T*_m_ and the enzymatic activity.

**Step 2 of GRAPE strategy: Clustering the stabilizing mutations**

Single point mutations usually enhance the stability marginally, but a much higher degree of stabilization can be achieved by combining beneficial mutations. Therefore, it is necessary to accumulate the experimentally validated beneficial mutations. Prior to the combination step, a clustering step by *K*-means algorithm (3) is performed to reduce the number of potential combination paths, with the cluster count set by users (default value is 3). Clustering parameters include Δ*T*_m_ improvements, locations of the Cα atoms, and the potential effects upon mutations. The changes in hydrogen bonding/hydrophobic interactions and conformational entropy are calculated automatically by Yasara (4) to inform the clustering parameters.

After clustering, users are recommended to accumulate the beneficial mutations in each cluster following the greedy strategy, starting with the mutation with the largest Δ*T*_m_ improvement. Subsequent mutations in the cluster are then combined with this parent mutation. This iterative process creates a new template each time, continuing until all mutations in the cluster have been assessed or the mutation combinations yield unsatisfactory results. Notably, the greedy accumulation step requires experimental screening to ensure the accurate exploration of epistatic effects. The systematic approach increases the likelihood of identifying adaptive routes to greater fitness with largely reduced experimental efforts.

**Supplementary Notes**

1. **Technical details of GRAPE-WEB**

In the local version of the GRAPE strategy, computational protocols that include extensive structure sampling can improve prediction accuracy but substantially slow down the computation speed, rendering it impractical for webserver applications. Based on user feedback, we have enhanced the web server's implementation, primarily focusing on speed improvement. After evaluating the time consumption and potential benefits of different tools, we opted for Rosetta_ddg_monomer algorithms, reducing the processing time from about two days to less than an hour on regular size proteins. For visualization, we employ Mol* for interactive structure inspection. Moreover, we offer an API along with detailed guidance for employing PyMOL for visual analysis, to facilitate a smoother user experience as extensive interactive options previously led to latency for some users. The front end is developed with the streamlit package, while the backend utilizes FastAPI and custom scripts to efficiently execute the GRAPE workflow. The GRAPE-WEB has been operating steadily for approximately two years.

1. **Mutation prediction software involved in GRAPE-WEB**

In GRAPE-WEB, we integrated FoldX, Rosetta, and ABACUS, each offering unique advantages. FoldX is an empirical force field-based method specifically designed for protein thermostability engineering and facilitated many experimental successes. Rosetta is a comprehensive protein modeling suite with a highly detailed energy function, widely used for protein design and stabilization. ABACUS is based on statistical potential that calculates conformational energies from the distribution of conformations in crystal structure data and is effective in protein sequence design and mutation effect prediction.

However, force field-based methods often suffer from inaccuracies in their energy functions and inadequate sampling of conformations, leading to less accurate predictions. Statistical potentials, meanwhile, rely on database data, which can result in biased likelihood estimations due to skewed training data.

To mitigate the biased predictions from each method and enrich as many beneficial mutations as possible, GRAPE-WEB combines stabilizing mutations predicted by each tool, leveraging both physical energy functions and statistical potentials. As shown in Table S5, we found that these three methods are relatively independent. For example, out of the 65 stabilizing mutations correctly predicted by FoldX and the 73 correctly predicted by ABACUS, only 18 overlapped. This confirms that combining these algorithms effectively enriches beneficial mutations.

1. **Criteria for visual inspection**

The following criteria are suggested for the visual inspection of the predicted mutant structures:

- 1. Hydrogen bonds and salt-bridge interactions:
     1. Mutations must not disrupt existing hydrogen bonds or salt-bridge interactions.
     2. No unsaturated hydrogen bonding interactions should be introduced, which could compromise structural stability.
     3. Use tools like YASARA to PyMOL or to identify and evaluate hydrogen bonds (typically within 2.5-3.5 Å) and salt-bridges (typically within 3.0-4.0 Å).
  2. Hydrophobic mutations:
     1. Hydrophobic mutations should not be positioned on the protein surface where they could interact unfavorably with the aqueous environment.
     2. Use hydropathy indices to ensure hydrophobic residues are buried within the protein core.
     3. Verify using YASARA or PyMOL to ensure these residues are not solvent-exposed or use structural software like freesasa or dssp to calculate the solvent exposure metrics.
  3. **Steric clashes and cavities creation**:
     1. Mutations should avoid steric clashes with surrounding residues.
     2. Check for van der Waals overlaps and ensure the minimum distance between non-bonded atoms is maintained. Avoid creating large cavities that can destabilize the protein.
     3. In YASARA, visualize protein in sphere mode can easily illustrate **clashes and cavities.**
  4. **Secondary structure stability**:
     1. Mutations should not destabilize the protein’s secondary structure, such as alpha helices and beta sheets. Therefore, Proline and Glycine are not allowed to present in a helix.
     2. Evaluate changes using Ramachandran plots to ensure phi (φ) and psi (ψ) angles fall within allowed regions for the mutated residues.

1. **The machine learning algorithm for mutation clustering**

*K*-means clustering employs *k* prototype vectors (centers or centroids of *k* clusters) to represent the data. It minimizes a sum-of-squares cost function through a coordinate descent optimization method to determine these prototypes. The algorithm uses Euclidean distance to measure the distances between instances and cluster centers. Given a set of individuals *x_1_*, *x_2_*, …, *x_n_*, *k*-means clustering seeks to divide these individuals into *k* (≤ *n*) sets. The objective function is defined as follows:

$$\begin{aligned} \arg min\sum_{i=1}^{k} \sum_{x\in S_{i}}^{k_{i}} \left\| x-\mu_{i} \right\|^{2}\# \end{aligned}$$

In this equation:

$\left\| x-\mu_{i} \right\|$ represents the Euclidean distance between the data point *x* and the centroid *µ_i_*.

*k_i_* denotes the number of individuals in the *i*th cluster.

*k* indicates the total number of clusters.

The features used for clustering include:

1. Experimentally measured ∆T_m_.
2. Changes in the number of hydrogen bonds formed with neighboring amino acids before and after the mutation, calculated using YASARA software.
3. Changes in hydrophobic contacts with neighboring amino acids before and after the mutation, also calculated using YASARA software.
4. Whether there is a significant change in entropy (assigned a value of 1 if the mutation involves Glycine or Proline, otherwise 0).
5. The Cartesian coordinates of the Cα atom where the mutation occurs.

The goal is to minimize the sum of squared distances between each data point and its corresponding cluster centroid.

1. **General procedure for the accumulation of individuals in each cluster by the greedy algorithm**

Greedy accumulation follows the problem-solving heuristic of making a locally optimal choice in each stage with the intent of finding a global optimum. Users can select the mutation with the highest Δ*T*_m_ as the starting point for the initial round of greedy accumulation. Then, the remaining mutations in the 1^st^ cluster are combined with the parents and resulted in a best hit in the second stage; the best hit is used as the new template for the next round of accumulation. Each round produces a best hit at the current stage and serves as the template for further accumulation. The combination process is repeated until the remaining mutations in the cluster are traversed or the *T*_m_ values of the combined variants decrease. For example, if a variant exhibits high thermostability but a degradation reduction of >50%, the combined variant is not adopted. The best result obtained in the 1^st^ cluster serves as the template for the next crossover mutation step in the 2^nd^ cluster. The exploitation process continues in a multihierarchical manner until all clusters are traversed.

1. **The case study of *Is*PETase highlighted the importance of the greedy accumulation**

Starting from 21 mutants displayed increased stability (∆*T*_m_ ≥1.5 °C), there would be approximately 10^19^ possible combination paths and 2 million combined mutations linking these beneficial single point mutations. We initially explored a stepwise combination of the most optimal single point mutations with Δ*T*_m_ ≥ 7 °C. A dramatic decrease in stability was obtained when combining I168R to S214H-D186H variant, which verified the importance of avoiding epistatic effects. However, with the greedy accumulation was experimentally performed to combine the single-point mutations in each cluster, the final mutant was gained with 65 experiment trails with significantly enhanced thermostability (Δ*T*_m_ = 31 °C). The final mutant also exhibited significantly enhanced degradation toward PET films by over 300-fold at mild temperatures.

For single point mutation prediction, both FireProt (1) and PROSS (2) exhibited similar predictive power that they identified 23 and 33 designed mutations, respectively. Among these mutations, only three specific mutations (L117F, W159H, and S214H) in DuraPETase were predicted by PROSS, with FireProt not predicting any of these mutations (Table S7).

1. **The case study of PAL demonstrated board applications and compatibility of GRAPE**

In the design of PAL for improving solvent tolerance, 62 mutants were characterized, and 17 demonstrated enhanced guanidinium chloride tolerance. While most beneficial mutations were derived from the predictions of Rosetta, FoldX, and ABACUS, an additional 4 beneficial mutations were identified by MSAddG, our newly developed machine learning-based predictor. MSAddG leverages evolutionary information from multiple sequence alignments (MSAs) and physicochemical embeddings to predict stability changes upon mutation. These results highlight the potential of machine learning methods to further enrich beneficial mutations within the GRAPE framework. However, they also indicate that the current ML-based predictor is not necessarily superior to traditional structure-based predictors. Indeed, we found that MSAddG, like other ML-based predictors, could be impaired by biases present in the training dataset and is sensitive to the quality of available MSAs. Consequently, we did not include it in GRAPE-WEB.

In our effort to develop a more robust variant with multiple mutations, our initial attempt to construct a mutant with all 17 mutations resulted in expression as inclusion bodies. To address the issues caused by negative epistatic interactions, we employed a greedy accumulation strategy previously proposed. This approach led to the creation of PAL14, which showed significant improvements over the wildtype. PAL14 exhibited increased denaturant tolerance, with half concentration values rising by 0.52 M for guanidinium chloride and 0.79 M for urea. Additionally, the ΔG value improved from −1.3 to −1.9 kcal/mol, reflecting enhanced thermodynamic stability. PAL14 also demonstrated a higher apparent *T*_m_ by 6.5 °C, indicating greater kinetic stability.

**Supplementary Tables**

**Table S1:** FoldX 5.0 results based on different threshold values

| Threshold | Accuracy | Precision | Specificity | Sensitivity | TP_counts | F1_score |
| --- | --- | --- | --- | --- | --- | --- |
| -2.5 | 0.776 | 0.654 | 0.996 | 0.028 | 17 | 0.054 |
| -2 | 0.777 | 0.612 | 0.991 | 0.050 | 30 | 0.092 |
| -1.5 | 0.784 | 0.650 | 0.983 | 0.108 | 65 | 0.185 |
| -1 | 0.786 | 0.598 | 0.965 | 0.178 | 107 | 0.274 |
| -0.5 | 0.793 | 0.587 | 0.936 | 0.307 | 185 | 0.403 |
| 0 | 0.775 | 0.505 | 0.844 | 0.540 | 325 | 0.522 |
| 0.5 | 0.708 | 0.420 | 0.697 | 0.746 | 449 | 0.537 |
| 1 | 0.623 | 0.362 | 0.553 | 0.862 | 519 | 0.510 |
| 1.5 | 0.532 | 0.316 | 0.421 | 0.907 | 546 | 0.468 |
| 2 | 0.465 | 0.291 | 0.325 | 0.942 | 567 | 0.445 |
| 2.5 | 0.409 | 0.273 | 0.247 | 0.958 | 577 | 0.424 |

**Table S2:** Rosetta_cartesian results based on different threshold values.

| Threshold | Accuracy | Precision | Specificity | Sensitivity | TP_counts | F1_score |
| --- | --- | --- | --- | --- | --- | --- |
| -2.5 | 0.792 | 0.691 | 0.979 | 0.156 | 94 | 0.255 |
| -2 | 0.797 | 0.692 | 0.975 | 0.194 | 117 | 0.304 |
| -1.5 | 0.805 | 0.703 | 0.969 | 0.248 | 149 | 0.366 |
| -1 | 0.810 | 0.676 | 0.955 | 0.319 | 192 | 0.433 |
| -0.5 | 0.810 | 0.633 | 0.933 | 0.395 | 238 | 0.487 |
| 0 | 0.808 | 0.591 | 0.897 | 0.503 | 303 | 0.543 |
| 0.5 | 0.795 | 0.544 | 0.847 | 0.620 | 373 | 0.579 |
| 1 | 0.776 | 0.506 | 0.799 | 0.701 | 422 | 0.588 |
| 1.5 | 0.742 | 0.459 | 0.734 | 0.767 | 462 | 0.575 |
| 2 | 0.702 | 0.419 | 0.673 | 0.802 | 483 | 0.551 |
| 2.5 | 0.666 | 0.391 | 0.615 | 0.839 | 505 | 0.533 |

**Table S3:** ABACUS results based on different threshold values.

| Threshold | Accuracy | Precision | Specificity | Sensitivity | TP_counts | F1_score |
| --- | --- | --- | --- | --- | --- | --- |
| -2.5 | 0.774 | 0.514 | 0.966 | 0.121 | 73 | 0.196 |
| -2 | 0.776 | 0.523 | 0.955 | 0.168 | 101 | 0.254 |
| -1.5 | 0.768 | 0.479 | 0.927 | 0.228 | 137 | 0.309 |
| -1 | 0.754 | 0.438 | 0.889 | 0.294 | 177 | 0.352 |
| -0.5 | 0.736 | 0.414 | 0.839 | 0.387 | 233 | 0.400 |
| 0 | 0.711 | 0.394 | 0.770 | 0.508 | 306 | 0.444 |
| 0.5 | 0.670 | 0.366 | 0.686 | 0.616 | 371 | 0.459 |
| 1 | 0.622 | 0.343 | 0.593 | 0.721 | 434 | 0.465 |
| 1.5 | 0.580 | 0.327 | 0.517 | 0.797 | 480 | 0.464 |
| 2 | 0.534 | 0.308 | 0.445 | 0.839 | 505 | 0.450 |
| 2.5 | 0.488 | 0.291 | 0.374 | 0.874 | 526 | 0.437 |

**Table S4:** GRAPE results based on different thresholds. The threshold values were chosen from -2.0 to -1.0 for FoldX and Rosetta with an interval of 0.5, and for ABACUS from -2.5 to -1.5 with an interval of 0.5, respectively.

| FoldX | Rosetta | ABACUS | Accuracy | Precision | Specificity | Sensitivity | TP_counts | F1_score |
| --- | --- | --- | --- | --- | --- | --- | --- | --- |
| -2 | -2 | -2.5 | 0.786 | 0.564 | 0.940 | 0.264 | 159 | 0.360 |
| -2 | -2 | -2 | 0.786 | 0.556 | 0.930 | 0.299 | 180 | 0.389 |
| -2 | -2 | -1.5 | 0.776 | 0.511 | 0.904 | 0.342 | 206 | 0.410 |
| -2 | -1.5 | -2.5 | 0.793 | 0.585 | 0.936 | 0.307 | 185 | 0.403 |
| -2 | -1.5 | -2 | 0.792 | 0.573 | 0.926 | 0.339 | 204 | 0.426 |
| -2 | -1.5 | -1.5 | 0.781 | 0.527 | 0.900 | 0.379 | 228 | 0.441 |
| -2 | -1 | -2.5 | 0.798 | 0.589 | 0.925 | 0.367 | 221 | 0.452 |
| -2 | -1 | -2 | 0.797 | 0.577 | 0.914 | 0.397 | 239 | 0.470 |
| -2 | -1 | -1.5 | 0.786 | 0.537 | 0.890 | 0.434 | 261 | 0.480 |
| -1.5 | -2 | -2.5 | 0.790 | 0.575 | 0.936 | 0.294 | 177 | 0.389 |
| -1.5 | -2 | -2 | 0.790 | 0.564 | 0.926 | 0.327 | 197 | 0.414 |
| -1.5 | -2 | -1.5 | 0.779 | 0.521 | 0.900 | 0.369 | 222 | 0.432 |
| -1.5 | -1.5 | -2.5 | 0.796 | 0.590 | 0.932 | 0.332 | 200 | 0.425 |
| -1.5 | -1.5 | -2 | 0.795 | 0.577 | 0.922 | 0.362 | 218 | 0.445 |
| -1.5 | -1.5 | -1.5 | 0.784 | 0.532 | 0.896 | 0.400 | 241 | 0.457 |
| -1.5 | **-1** | **-2.5** | **0.799** | **0.590** | **0.921** | **0.385** | **232** | **0.466** |
| -1.5 | -1 | -2 | 0.798 | 0.578 | 0.911 | 0.414 | 249 | 0.482 |
| -1.5 | -1 | -1.5 | 0.787 | 0.539 | 0.887 | 0.450 | 271 | 0.490 |
| -1 | -2 | -2.5 | 0.789 | 0.559 | 0.921 | 0.339 | 204 | 0.422 |
| -1 | -2 | -2 | 0.788 | 0.550 | 0.912 | 0.367 | 221 | 0.440 |
| -1 | -2 | -1.5 | 0.776 | 0.511 | 0.886 | 0.404 | 243 | 0.451 |
| -1 | -1.5 | -2.5 | 0.792 | 0.567 | 0.917 | 0.367 | 221 | 0.446 |
| -1 | -1.5 | -2 | 0.791 | 0.556 | 0.908 | 0.394 | 237 | 0.461 |
| -1 | -1.5 | -1.5 | 0.779 | 0.516 | 0.882 | 0.427 | 257 | 0.467 |
| -1 | -1 | -2.5 | 0.793 | 0.564 | 0.908 | 0.405 | 244 | 0.471 |
| -1 | -1 | -2 | 0.792 | 0.554 | 0.898 | 0.432 | 260 | 0.486 |
| -1 | -1 | -1.5 | 0.781 | 0.519 | 0.873 | 0.465 | 280 | 0.491 |

**Table S5:** Number of overlaps of the correctly predicted stabilizing mutations by FoldX, Rosetta, and ABACUS. Thresholds values were chosen based on the results captured in Table 1, the FoldX, Rosetta, and ABACUS thresholds were set to -1.5 kcal/mol, -1.0 REU, and -2.5 AEU, respectively.

| Algorithm | FoldX_TP | Rosetta_TP | ABACUS_TP |
| --- | --- | --- | --- |
| FoldX_TP | 65 | 50 | 18 |
| Rosetta_TP | 50 | 192 | 48 |
| ABACUS_TP | 18 | 48 | 73 |

**Table S6:** Clustering results for the 21 identified stabilizing mutations of IsPETase

| Cluster | Stabilizing mutations |
| --- | --- |
| Cluster 1 | K95A, W159H, I168R, D186H, S188Q, S214H, A248P, and R280A |
| Cluster 2 | L117F, Q119Y, G165A, S166T, A180I, P181A, S187W, I208V, N212R, and S238F |
| Cluster 3 | T77E, T140D, and K148W |

**Table S7:** Prediction of stabilizing mutations of IsPETase by FireProt and PROSS^a^

| Mutations | Δ*T*_m_ | FireProt | PROSS | Mutations | Δ*T*_m_ | FireProt | PROSS |
| --- | --- | --- | --- | --- | --- | --- | --- |
| N37P | -0.5 |  | D | Q182L | -2.5 | L | L |
| A40P | -0.5 |  | E | A183T | -5 |  |  |
| A47W | -0.5 | R | R | D186H | 7 | N | H |
| T51P | -1.5 | A | A | D186W | -1 |  |  |
| S54W | -1.5 | Y |  | S187P | -1.5 | P | T |
| G66A | -0.5 |  |  | S187W | 3 |  |  |
| A74T | -3.5 |  |  | S188Q | 1.5 |  |  |
| T77E | 1.5 |  |  | T189K | -1.5 | K |  |
| T77P | -1 | P | P | S193R | -0.5 |  | E |
| A82V | -6.5 |  | V | T198V | 0 |  |  |
| R90D | -1 |  | T | E204K | -0.5 |  | Q |
| Q91P | -8.5 |  |  | I208V | 1.5 | W |  |
| S92P | -2 |  |  | P210K | -1.5 |  |  |
| K95A | 2.5 | A | A | N212R | 3 |  |  |
| R100F | -3 |  |  | S214H | 9 |  | Y |
| H104F | -1.5 |  |  | A215P | -6 |  |  |
| D112R | -3 |  |  | P217L | -0.5 |  |  |
| T113P | -3.5 |  |  | I218F | -8.5 |  | M |
| T116P | 0.5 |  | L | D220E | -0.5 |  | N |
| L117F | 3 |  | Y | S223P | -6.5 |  |  |
| Q119Y | 4.5 |  |  | N225V | -6 |  |  |
| S121D | -1.5 |  | D | A226P | -1 |  |  |
| R123V | -6 |  |  | Q228V | -8.5 | A |  |
| S125R | 0 | R | R | E231M | 0.5 |  |  |
| Q133Y | -3.5 | Y |  | E231R | -4.5 |  |  |
| V134L | 1 | L | L | S238F | 1.5 | F | Y |
| S136Q | -0.5 | W | Q | A240V | 0.5 |  |  |
| G139N | 1 |  | N | A240P | 0.5 |  |  |
| T140D | 2 |  |  | N241L | -1.5 | T | T |
| S141P | 1 | P |  | N246W | -6 | W | H |
| G147T | 1 |  |  | A248P | 3 |  |  |
| K148W | 2 | R |  | K253Y | 0.5 |  |  |
| T151P | 1 |  |  | G254V | -4 |  | A |
| A152S | 0 |  | S | K259F | -2 |  |  |
| M154F | 0 |  |  | T266P | -2.5 |  |  |
| G155A | 0.5 |  |  | T270Q | -2.5 | Q | Q |
| W159H | 8.5 |  | H | A272L | 0 |  |  |
| G165A | 1.5 |  |  | E274P | 1 |  | A |
| S166T | 2 |  | A | T279P | 0.5 | S | P |
| I168R | 7.5 |  |  | R280A | 1.5 |  |  |
| S175W | 0 | W |  | T286V | -4.5 |  |  |
| A180I | 1.5 |  |  | S290P | 0.5 |  |  |
| P181A | 6 |  |  |  |  |  |  |

^a^ Mutations marked in red represent that they are involved in DuraPETase

**Supplementary Figures**


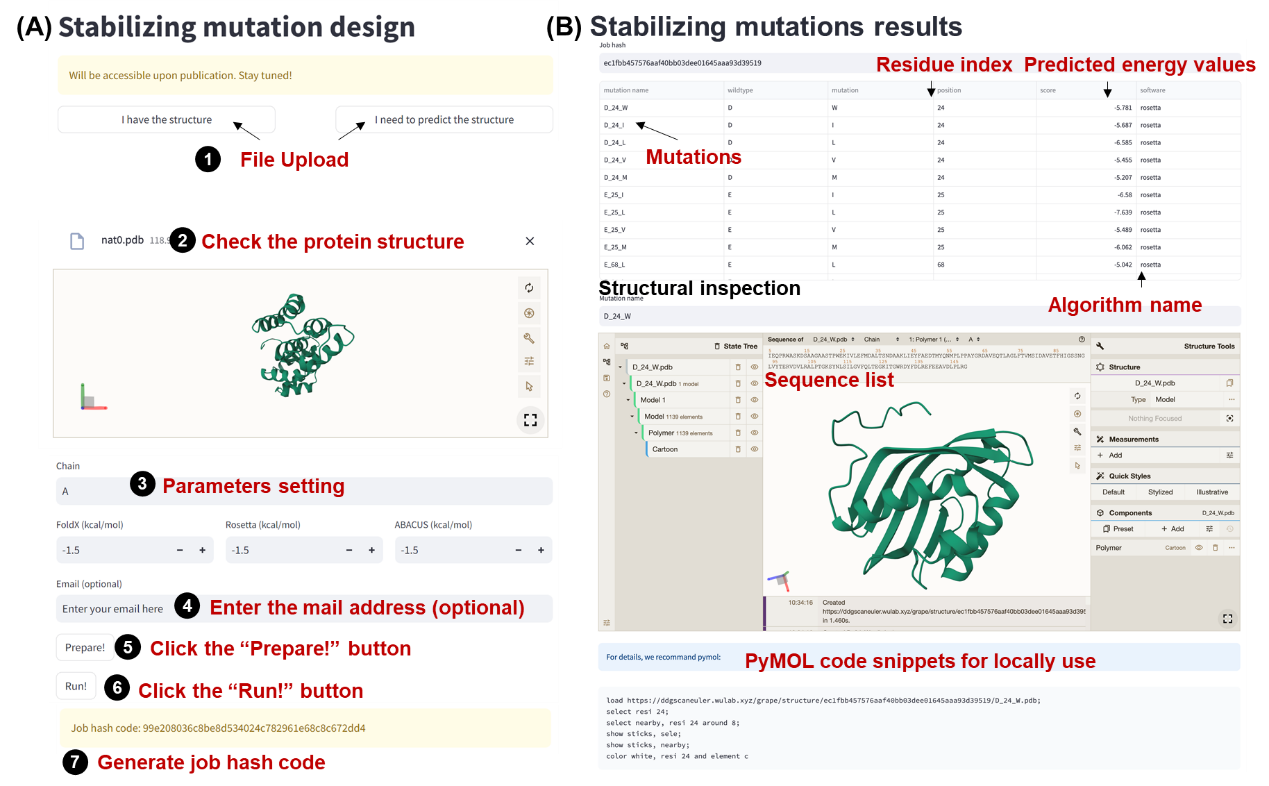


**Figure S1.** User interface of the GRAPE-WEB server for single point mutation design. Step 1 of GRAPE strategy: Design of stabilizing mutations. (A) The ‘Stabilizing mutation design’ panel allows users to design stabilizing single-point mutations using the hybrid design approach. (B) An example of the design results of a limonene-1,2-epoxide hydrolase (PDB ID: 1NWW). The table provides the mutations information and their predicted energies. The Mol* interface allows visualization of the mutations.


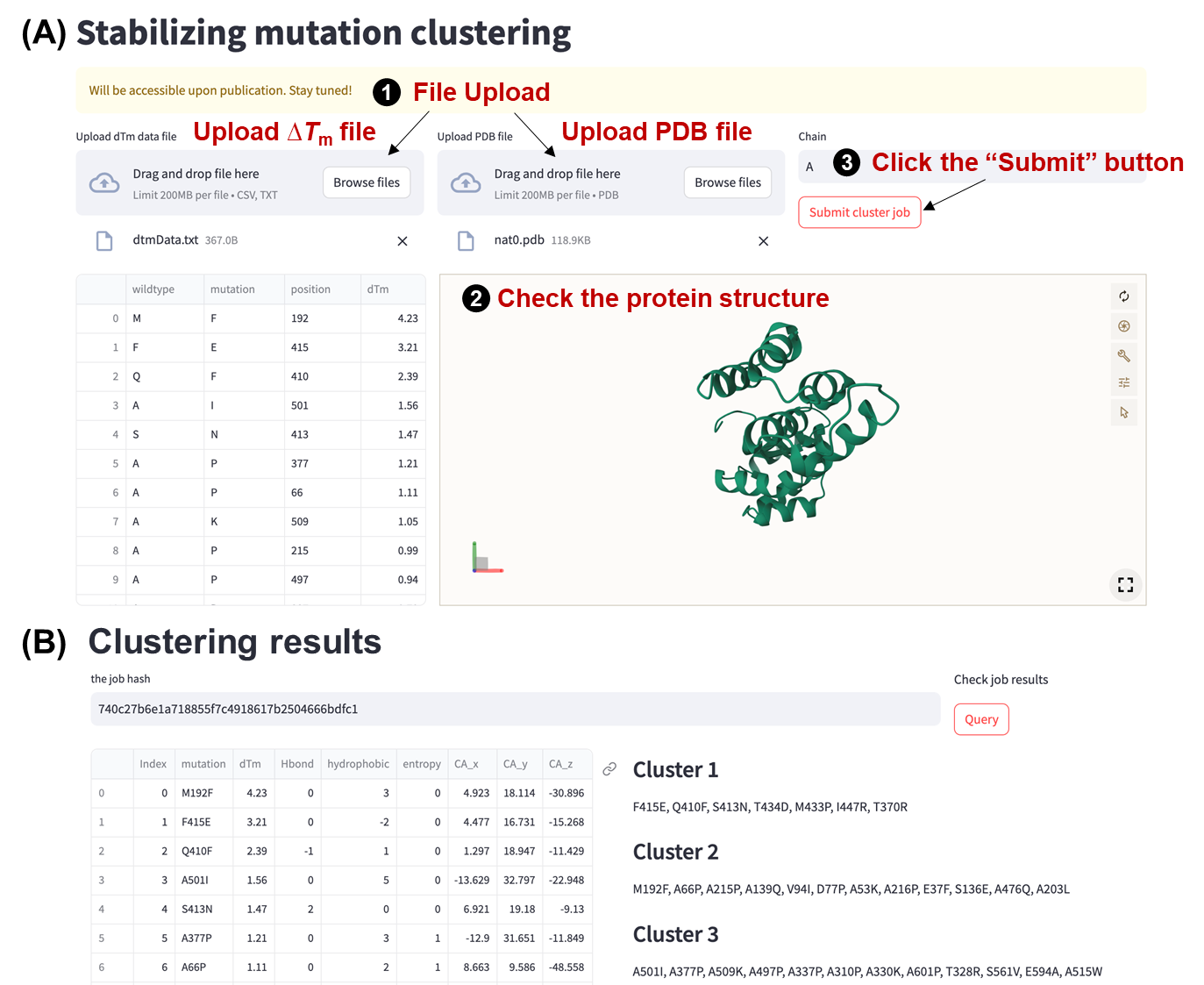


**Figure S2.** User interface of the GRAPE-WEB server for mutation clustering. Step 2 of GRAPE strategy: Clustering the stabilizing mutations. (A) The ‘Stabilizing mutations clustering’ panel allows users to cluster the experimentally validated beneficial mutations to reduce the number of combination paths. (B) An example of clustering results of beneficial mutations. The left table provides the clustering features used in the calculations, including the beneficial mutations information and the potential effects caused by the mutations. The right table provides the clustering results.

**References:**

1. Park H, Bradley P, Greisen-Jr P, Liu Y, Mulligan VK, Kim DE, et al. Simultaneous optimization of biomolecular energy function on features from small molecules and macromolecules. *J Chem Theory Comput.* 2016; 12(12): 6201-6212. <https://doi.org/10.1021/acs.jctc.6b00819>
2. Eastman P, Swails J, Chodera JD, McGibbon RT, Zhao Y, Beauchamp KA, et al. OpenMM 7: Rapid development of high performance algorithms for molecular dynamics. *PLoS Comput. Biol.*, 2017, 13(7): e1005659. <https://doi.org/10.1371/journal.pcbi.1005659>
3. Jain AK. Data clustering: 50 years beyond K-means. *Pattern Recognit Lett.* 2010; 31: 651-666. <https://doi.org/10.1016/j.patrec.2009.09.011>
4. Krieger E and Vriend G. New ways to boost molecular dynamics simulations. *J Comput Chem.* 2015; 36(13): 996-1007. <https://doi.org/10.1002/jcc.23899>
5. Musil M, Stourac J, Bendl J, Brezovsky J, Prokop Z, Zendulka J, et al. FireProt: web server for automated design of thermostable proteins. *Nucleic Acids Res.* 2017; 45(W1): W393-W399. <https://doi.org/10.1093/nar/gkx285>
6. Goldenzweig A, Goldsmith M, Hill SE, Gertman O, Laurino P, Ashani Y, et al. Automated structure-and sequence-based design of proteins for high bacterial expression and stability. *Mol Cell.* 2016; 63(2): 337-346. <https://doi.org/10.1016/j.molcel.2016.06.012>
